# Central obesity in psoriatic arthritis: associations with disease activity, function, and quality of life in a real-world cohort

**DOI:** 10.3389/fmed.2025.1684641

**Published:** 2025-10-02

**Authors:** Salvatore Corrao, Salvatore Scibetta, Luigi Calvo, Marika Lo Monaco, Raffaella Mallaci Bocchio, Giuseppe Natoli, Antonella Montalbano, Ignazio Cangemi, Christiano Argano

**Affiliations:** ^1^Department of Health Promotion Sciences, Maternal and Infant Care, Internal Medicine and Medical Specialties [PROMISE], University of Palermo, Palermo, Italy; ^2^Department of Clinical Medicine, Internal Medicine Unit with Rheumatology, Dermatology, Diabetology and Tertiary Diabetic Foot Health-care, National Relevance and High Specialization Hospital Trust ARNAS Civico, Di Cristina, Benfratelli, Palermo, Italy; ^3^Institute for Biomedical Research and Innovation (IRIB), National Research Council (CNR), Palermo, Italy

**Keywords:** psoriatic arthritis, central obesity, disease activity, quality of life, patient-reported outcomes, inflammation

## Abstract

**Objective:**

This study aimed to explore the association between central obesity and disease-specific activity, functional impairment, and patient-reported outcomes (PROMs) in a real-world cohort of individuals with psoriatic arthritis (PsA).

**Methods:**

We conducted a cross-sectional observational study at a tertiary rheumatology outpatient clinic. Adult patients with PsA, diagnosed according to the CASPAR criteria, underwent a multidimensional assessment by a multidisciplinary team. Clinical and pharmacological data, anthropometric measures, disease activity indices (DAPSA and CRP), and PROMs (FACIT-F, PHQ-9, HAQ, PsAID) were collected. Central obesity was defined by waist circumference (≥88 cm in women, ≥102 cm in men). Multivariable regression analyses were performed to investigate associations between central obesity and clinical outcomes, adjusting for age, sex, disease duration, and antirheumatic therapies.

**Results:**

Among 158 patients (54.4% female; median age 55.4 years), central obesity was present in 70.2% of the group. Patients with central obesity had significantly higher inflammatory markers (CRP), increased disease activity (DAPSA), and worse scores across all PROMs compared to those without. In multivariable models, central obesity remained independently linked to disease activity and worse physical and psychosocial outcomes, including fatigue, depression, disability, and disease impact.

**Conclusion:**

Central obesity is a significant and independent predictor of disease severity and patient-reported disability in individuals with Psa. These findings highlight the clinical importance of routinely measuring waist circumference in PsA management and suggest that targeted interventions addressing visceral adiposity could enhance both inflammatory and quality-of-life outcomes.

## Introduction

Psoriatic arthritis (PsA) is a chronic, immune-mediated inflammatory musculoskeletal disorder linked with psoriasis that leads to joint damage and functional decline (1). Historically, epidemiological studies on PsA have faced difficulties due to the lack of widely accepted classification criteria (2). Nevertheless, PsA has been consistently recognized as a common and significant comorbidity in patients with psoriasis, affecting between 5 and 20% of this population (3). Its prevalence seems to be rising globally, especially among older individuals (1).

Beyond joint involvement, PsA is now recognized as a systemic disease with a wide range of extra-articular manifestations and comorbidities. Notably, patients often display a higher incidence of autoimmune thyroid disorders and a tendency toward thyroid dysfunction, reflecting heightened immune activation compared to individuals with psoriasis alone (4). Furthermore, PsA has been associated with an increased risk of severe vascular complications, including cardiovascular disease (5), as well as comorbidities such as uveitis, fibromyalgia, osteoporosis, Crohn’s disease, non-alcoholic fatty liver disease, and metabolic syndrome (6–8). The impact of the disease also extends to mental health: anxiety and depression are more common among PsA patients and are linked to the extent of skin involvement, significantly impairing quality of life (9).

The implementation of the Classification Criteria for Psoriatic Arthritis (CASPAR) has enhanced diagnostic accuracy and supported research in this area (10). These criteria combine clinical, radiological, and immunological variables to identify PsA with high sensitivity and specificity (11, 12).

Obesity has emerged as a significant factor in the epidemiology and clinical progression of Psa. Numerous studies have emphasized its connection with disease incidence, severity, and treatment outcomes. Husted et al. reported that 30% of PsA patients have obesity (13), while Klingberg and colleagues confirmed a strong link between obesity, metabolic syndrome, and PsA disease burden (14, 15). Furthermore, obesity has been associated with decreased treatment efficacy and increased cardiovascular risk (15). Jensen and Skov identified obesity as a risk factor for the development of psoriasis (16), which may subsequently lead to PsA, whereas Kovitwanichkanont et al. demonstrated that obesity is an independent risk factor for PsA in individuals with psoriasis (17). These findings emphasize the importance of weight management in this patient population. Dietary interventions, such as hypocaloric regimens, have shown promise in reducing disease activity and enhancing quality of life (18).

Nevertheless, the traditional definition of obesity based solely on body mass index (BMI) may not adequately reflect metabolic risk. Central (abdominal) obesity—defined by excess visceral fat—is increasingly recognized as a more accurate predictor of cardiovascular and metabolic morbidity (19). This pattern of fat distribution is linked to a higher risk of insulin resistance, type 2 diabetes, and cardiovascular events.

Recent research has examined the potential role of central obesity in PsA pathogenesis. Gelfand et al. (20) found a significant prevalence of obesity among PsA patients, supporting its influence on disease severity and cardiovascular risk. The authors also noted the large number of individuals at risk for PsA among those with psoriasis, especially those with central obesity (21). This link was further supported by findings from Kovitwanichkanont et al. and Li et al., who emphasized the pathogenic role of visceral adiposity to PsA development and progression of Psa (17, 22).

Given this data, it is important to examine not only general obesity but also central fat distribution in PsA patients. Our study aimed to evaluate the association between central obesity and disease-specific activity, functional status, and quality of life in a real-world cohort of individuals affected by PsA.

## Methods

A cross-sectional observational study was conducted at the Rheumatology Outpatient Clinic of the Internal Medicine Department, National Relevance and High Specialization Hospital Trust ARNAS Civico, Di Cristina, Benfratelli, Palermo, Italy. The study was approved by the Local Ethics Committee (Protocol No. 231 CIVICO 2018) and carried out in accordance with the principles outlined in the Declaration of Helsinki and its later amendments. All the patients signed an informed consent for data treatment for research purposes.

### Study population

Adult patients (≥18 years) with a diagnosis of psoriatic arthritis (PsA) fulfilling the CASPAR (Classification Criteria for Psoriatic Arthritis) criteria for peripheral involvement were consecutively recruited (10–12). Participants were enrolled following the provision of written informed consent, without additional exclusion criteria, to ensure the inclusion of a real-world clinical population.

### Multidisciplinary evaluation

Each patient underwent a comprehensive assessment carried out by an interprofessional team, including rheumatologists, nurse specialists, and a clinical nutritionist. Disease activity was measured using the Disease Activity in Psoriatic Arthritis (DAPSA) score and serum C-reactive protein (CRP). Joint involvement was evaluated through 66/68 swollen and tender joint counts. A nutritionist took anthropometric measurements. Waist circumference was used to define central obesity, applying the internationally accepted thresholds of ≥88 cm for women and ≥102 cm for men (23). Body mass index (BMI) was also calculated for descriptive purposes.

### Patient-reported outcomes and additional data

Patient-reported outcomes (PROMs) were assessed using standardized, validated instruments: fatigue was measured by the Functional Assessment of Chronic Illness Therapy–Fatigue Scale (FACIT-F), mood via the Patient Health Questionnaire-9 (PHQ-9), and functional status by the Health Assessment Questionnaire (HAQ). The Psoriatic Arthritis Impact of Disease (PsAID) questionnaire was utilized to evaluate the impact of PsA on quality of life. Nurse specialists gathered additional sociodemographic and behavioral data, including educational level, smoking and alcohol consumption, caregiving status, and family structure. These data were collected during routine clinical encounters, which enhances the ecological validity of the study.

### Statistical analysis

Descriptive statistics were presented as medians and interquartile ranges (IQR) for continuous variables, and percentages for categorical data. The Mann–Whitney U test was utilized to compare continuous variables between groups with and without central obesity, while Fisher’s exact test or the z-test for proportions was applied for categorical comparisons. Multivariable regression models were developed to explore the independent association between central obesity and outcome measures, including disease activity indices (DAPSA, CRP) and PROMs (FACIT-F, PHQ-9, HAQ, PsAID). Covariates in the models included age, gender, disease duration, and the use of conventional synthetic (csDMARDs) and biological disease-modifying antirheumatic drugs (bDMARDs). Odds ratios (ORs) and 95% confidence intervals (CIs) were computed for each association. A *p*-value <0.05 was regarded as statistically significant. All statistical analyses were performed using Stata Statistical Software: Release 18 (StataCorp LLC, College Station, TX, USA).

## Results

A total of 158 patients diagnosed with psoriatic arthritis (PsA) were included in the analysis. The median age was 55.4 years (IQR 47.1–63.7), and 54.4% of participants were women. Central obesity, defined by waist circumference thresholds (≥88 cm in women; ≥102 cm in men) (23), was present in 70.2% of the study population. The prevalence of central obesity was higher among women (77.9%) than men (61.1%).

**Figure 1:**
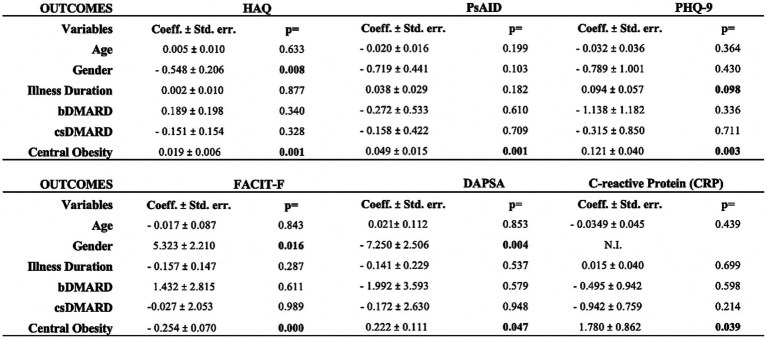
Multivariable analyses according to various outcome variables. csDMARDs, conventional synthetic disease-modifying anti-rheumatic drugs; bDMARDs, biological disease-modifying anti-rheumatic drugs; DAPSA, Disease Activity Index for Psoriatic Arthritis; FACIT-F, Functional Assessment of Chronic Illness Therapy – Fatigue; PHQ-9, Patient Health Questionnaire-9; HAQ, Health Assessment Questionnaire; PSAID, Psoriatic Arthritis Impact of Disease; PASI, Psoriasis Area Severity Index.

The median body mass index (BMI) for the entire sample was 28.1 kg/m^2^ (IQR 25.2–31.2), with those affected by central obesity showing significantly higher BMI values (median 29.4 vs. 24.4 kg/m^2^, *p* < 0.0001). Demographic and clinical characteristics stratified by central obesity status are presented in Tables 1, 2. Patients with central obesity were significantly older than those without (median age 57.9 vs. 50.4 years; *p* = 0.0011), and more frequently female (*p* = 0.0214). In terms of disease activity, patients with central obesity exhibited higher DAPSA scores (median 19.2 vs. 16.4; *p* = 0.0751) and significantly elevated CRP levels (median 1.92 vs. 0.57 mg/L; *p* = 0.0019). Swollen joint counts were significantly greater among those with central obesity (median 1.12 vs. 0.5; *p* < 0.0001), whereas tender joint counts were paradoxically lower (*p* = 0.0010). Regarding patient-reported outcomes, individuals with central obesity reported significantly worse scores across all domains. Fatigue, as measured by FACIT-F, was more pronounced in the obese group (median 30 vs. 39; *p* = 0.0010, lower scores indicating more fatigue). Similarly, PHQ-9 scores revealed greater depressive symptoms (median 8 vs. 4; *p* = 0.0063), and HAQ scores showed increased disability (median 1.12 vs. 0.5; *p* < 0.0001). Quality of life was significantly impaired among patients with central obesity, as indicated by higher PsAID scores (median 4.45 vs. 2.25; *p* = 0.0010). Multivariable regression models, adjusted for age, gender, disease duration, and DMARD therapy, confirmed central obesity as an independent predictor of higher DAPSA and CRP levels, as well as all PROMs evaluated (Figure 1). In contrast, gender showed a selective effect: female sex was independently associated with higher DAPSA and HAQ scores, while male sex correlated with higher fatigue (lower FACIT-F).

**Table 1:** Demographic and clinical characteristics of our population sample.

Variables	Values
No	158
Women (%)	54.4
Age	55.5 (47.1–63.6)
Age men	56.2 (47.2–63.6)
Age women	55.4 (47.1–63.7)
Disease duration	10.6 (9.3–11.9)
Subjects without caregivers (%)	38.6
Education (%)	
Elementary/Secondary school	15. 4
High school	31
Graduation	9
Family members	3 (2–4)
Smokers (%)	24.7
Alcohol consumption (%)	5.7
CASPAR Criteria	5 (4–6)
BMI	28.9 (28–29.8)
Waist Circumference	104.8 (102.6–107)
Waist Circumference men	105.5 (102.5–108.5)
Waist Circumference women	104.3 (101–107.5)
Central Obesity (%)	70.2
Central Obesity men (%)	61.1
Central Obesity women (%)	77.9
csDMARDs (%)	41.6
bDMARDs (%)	81.3
Combination of csDMARDs and bDMARDs (%)	74.5
DAPSA	19.0 (9–33.1)
CRP (mg/l)	1.47 (0.3–3.0)
66 Swollen Joint Count	2 (0–6)
68 Tender Joint Count	4 (0–13)
Facit F	35 (20–43)
PHQ-9	7 (4–13)
HAQ	1 (0.2–1.7)
PSAID	4 (2–6)
PASI	1 (0–2)
VAS	

Data are reported as median (interquartile range Q1-Q3).

CASPAR, Classification for Psoriatic Arthritis Criteria; BMI, Body Mass Index; csDMARDs, conventional synthetic disease-modifying anti-rheumatic drugs; bDMARDs, biological disease-modifying anti-rheumatic drugs; DAPSA, Disease Activity Index for Psoriatic Arthritis; CRP: C-reactive Protein; FACIT-F, Functional Assessment of Chronic Illness Therapy – Fatigue; PHQ-9, Patient Health Questionnaire-9; HAQ, Health Assessment Questionnaire; PSAID, Psoriatic Arthritis Impact of Disease; PASI, Psoriasis Area Severity Index, VAS, Visual Analogue Scale.

**Table 2:** Differences in demographic and clinical variables according to central obesity categorization.

Variables	Patients with central obesity (*n* = 111)	Patients without central obesity (*n* = 47)	*p*-values
Women (%)	60.4	40.4	**0.0214**
Age	57.9 (49.3–65.5)	50.4 (42.3–59.0)	**0.0011**
Age men	60.3 (50.3–65.5)	54.2 (41.8–60.6)	**0.0266**
Age women	57.9 (48.7–65.5)	49.4 (42.3–55.2)	**0.0119**
Disease duration	10 (4–15)	10 (2–14)	0.4623
Subjects without caregivers (%)	29.7	59.6	**0.0004**
Education (%)			
Elementary/Secondary school	61.3	55.3	0.4867
High school	29.7	34.0	0.5921
Graduation	8.1	10.6	0.6089
Family members	3 (2–4)	3 (2–4)	0.1282
Smokers (%)	23.4	27.7	0.5724
Alcohol Consumption (%)	7.3	2.1	0.2041
CASPAR Criteria	5 (4–6)	5 (4–5)	0.1638
BMI	29.4 (27.5–33.2)	24.4 (22.0–26.4)	**<0.0001**
csDMARDs (%)	36.0	29.8	0.4490
bDMARDs (%)	78.3	90.2	0.0974
Combination of csDMARDs and bDMARDs (%)	24.3	23.4	0.9016
DAPSA	19.2 (10.5–35.4)	16.4 (6.24–26.2)	0.0751
CRP (mg/l)	1.92 (0.5–3.9)	0.6 (0.20–2.0)	**0.0019**
66 Swollen Joint Count	1.1 (0.5–2)	0.5 (0–1.12)	**<0.0001**
68 Tender Joint Count	30 (20–39)	39 (32–47)	**0.0010**
FACIT-F	30 (20–39)	39 (32–47)	**0.0010**
PHQ-9	8 (5–14)	4 (2–12)	**0.0063**
HAQ	1.1 (0.5–2)	0.5 (0.0–1.12)	**<0.0001**
PsAID	4.4 (2.6–6.15)	2.2 (0.85–4.95)	**0.0010**
PASI	1.5 (0.0–2.7)	0.0 (0.0–1.2)	**0.0023**
VAS	50 (20–70)	40 (10–70)	0.1557

Data are reported as median (interquartile range Q1-Q3).

N.B. CASPAR Criteria, Classification for Psoriatic Arthritis Criteria; BMI, Body Mass Index; csDMARDs, conventional synthetic disease modifying anti-rheumatic drugs; bDMARDs, biological disease-modifying anti-rheumatic drugs; DAPSA, Disease Activity Index for Psoriatic Arthritis; CRP, C-reactive Protein; FACIT-F, Functional Assessment of Chronic Illness Therapy – Fatigue; PHQ-9, Patient Health Questionnaire-9; HAQ, Health Assessment Questionnaire; PSAID, Psoriatic Arthritis Impact of Disease; PASI, Psoriasis Area Severity Index, VAS, Visual Analogue Scale.

Significant *p*-values are reported in bold ones.

These findings suggest that central obesity not only clusters with systemic inflammation, but also significantly contributes to perceived disease burden and functional impairment, consistent with prior evidence linking visceral adiposity to inflammatory cytokine activation.

## Discussion

Psoriatic arthritis (PsA) is increasingly recognized as a systemic inflammatory disease with a significant comorbid burden, including metabolic, cardiovascular, and psychological components (5, 6, 24, 25). In this real-world cross-sectional study, we show that central obesity is highly common among PsA patients and is independently linked to both objective disease activity and subjective health outcomes. These findings support the hypothesis that visceral adiposity is a clinically important contributor to the disease progression and patient experience in PsA.

Our results show that over 70% of PsA patients exhibited central obesity, with significantly higher prevalence in women. This is in line with previous epidemiological research indicating a strong link between obesity and PsA incidence and severity (13–15). Importantly, we observed that central obesity was independently associated with elevated CRP and DAPSA scores highlighting its relevance as a marker of systemic inflammation.

Furthermore, patients with central obesity reported significantly worse fatigue, mood disturbances, functional impairment, and overall disease impact, as measured by FACIT-F, PHQ-9, HAQ, and PsAID scores. These associations persisted after adjusting for confounders, supporting central obesity as an independent predictor of poor patient-reported outcomes. This aligns with previous studies indicating that obesity adversely affects treatment response and quality of life in PsA (14, 15, 17, 18, 25).

It is noteworthy that while CRP levels and swollen joint counts were significantly higher in patients with central obesity, DAPSA scores did not reach statistical significance. This apparent discrepancy may be explained by the paradoxical reduction in tender joint counts observed in obese individuals, as DAPSA is a composite index that combines both swollen and tender joints along with patient-reported measures. These findings suggest that central obesity may affect pain perception or reporting, thereby reducing composite disease activity scores despite objective evidence of increased inflammation. Moreover, this discrepancy may have masked the positive relationship between central obesity and PsA outcomes; nonetheless, the association was clearly evident across multiple objective measures and patient-reported data. A plausible biological explanation lies in the complex interplay between obesity, chronic pain, and aging, all of which represent major global health challenges. Central to these processes are adrenal hormones, particularly cortisol and dehydroepiandrosterone (DHEA) with its sulfated form (DHEAS). Cortisol, while essential for stress adaptation, may negatively influence pain perception and accelerate aging when dysregulated, whereas DHEA/S exert counteracting properties that can mitigate these effects. Such hormonal dynamics may partially explain the paradoxical attenuation of pain perception in obese individuals, contributing to lower tender joint counts despite evidence of increased systemic inflammation (26).

Furthermore, the link between central obesity and worse patient-reported outcomes (fatigue, depression, disability, and disease impact) is probably mediated by multiple, interconnected pathways. Biologically, visceral adipose tissue is metabolically active, releasing pro-inflammatory adipokines and cytokines, including leptin, resistin, TNF-*α*, and IL-6, while decreasing protective adiponectin. This profile sustains systemic inflammation, contributes to fatigue, and impairs physical function in PsA patients (27, 28). Intervention studies show that weight loss leads to reductions in inflammatory mediators, most consistently IL-23, alongside improvements in disease activity (29). Clinically, structured weight loss has been linked to significant improvements in health-related quality of life and decreases in anxiety and depression (30). Moreover, patients meeting criteria for metabolic syndrome display greater pain catastrophizing, indicating that metabolic dysregulation influences pain perception and its impact in PsA (31). Overall, these findings support a multidimensional model in which central obesity exacerbates PsA through both inflammatory adipokine signaling and psychosocial amplification of symptoms. The connection between central obesity and inflammation may also be mediated by visceral adipose tissue, which is known to be metabolically active and pro-inflammatory. Visceral fat releases adipokines and cytokines such as TNF-*α*, IL-6, and resistin, contributing to chronic low-grade inflammation, endothelial dysfunction, and immune dysregulation (32–37). Fontana et al. demonstrated that visceral fat is a significant source of systemic inflammatory cytokines in obese individuals (38), while Matsuzawa highlighted the inverse relationship between visceral fat and adiponectin levels, suggesting an anti-inflammatory imbalance (39). These mechanisms may directly enhance psoriatic inflammation and indirectly exacerbate comorbidities such as cardiovascular disease.

Our findings have important clinical implications. Firstly, waist circumference should be routinely measured in PsA patients as part of comprehensive disease management. Secondly, strategies to reduce weight, especially targeting visceral fat, may provide dual benefits: lowering systemic inflammation and enhancing quality of life. Although some interventional studies suggest that weight loss can improve PsA disease activity and cardiovascular risk (14, 15, 18), the exact impact of reducing central obesity on PROMs remains insufficiently studied.

The strengths of this study include its real-world setting, thorough assessment by a multidisciplinary team, and the use of validated outcome measures. However, limitations must be recognized. The cross-sectional design prevents causal inference, and the study was conducted at a single center, which may limit generalisability. Therefore, our results should be considered as associations between central obesity and PsA outcomes rather than direct effects. Future longitudinal cohort studies and interventional trials specifically targeting visceral adiposity are needed to determine whether central obesity directly influences PsA severity and patient-center outcomes. Additionally, the lack of detailed dietary or physical activity data limits the interpretation of lifestyle effects. Despite these limitations, our findings emphasize the importance of addressing central obesity as a modifiable factor in PsA management. An interdisciplinary approach involving rheumatologists, nurses, nutritionists, and mental health professionals is vital to optimize outcomes in this complex patient group.

In conclusion, central obesity is a common and clinically significant condition in patients with psoriatic arthritis. It is independently linked to increased disease activity, heightened systemic inflammation, and notably poorer patient-reported outcomes. These findings imply that targeted interventions focusing on abdominal adiposity could be crucial for enhancing both inflammatory control and patient-centered care in PsA.

## Data Availability

The raw data supporting the conclusions of this article will be made available by the authors, without undue reservation.
